# Fearful snake pictures make monkeys pessimistic

**DOI:** 10.1016/j.isci.2023.107622

**Published:** 2023-08-12

**Authors:** Sakumi Iki, Ikuma Adachi

**Affiliations:** 1 Center for the Evolutionary Origins of Human Behavior, Kyoto University, Inuyama, Aichi, Japan; 2 Japan Society for the Promotion of Science, Tokyo, Japan

**Keywords:** Natural sciences, Biological sciences, Behavioral neuroscience

## Abstract

Judgment bias is the cognitive tendency of animals experiencing negative (or positive) affect to expect undesirable (or favorable) outcomes in ambiguous situations. The lack of examination of judgment biases induced by ecologically relevant stimuli hampers our understanding of the adaptive role of these biases. We examined whether predator-related stimuli, i.e., pictures of snakes, induce a pessimistic judgment bias in Japanese macaques (*Macaca fuscata*). Our subjects underwent a touchscreen-based Go/No-go judgment bias test. We found that the subjects were less likely and slower to make Go responses to ambiguous stimuli after viewing the snake pictures, indicating that pictures of snakes induce a pessimistic evaluation of ambiguous stimuli. In environments with high levels of threat, behavioral strategies that reduce risk-taking would be evolutionarily advantageous. Hence, an affective response system that lowers expectations of favorable outcomes in ambiguous situations after encountering threat-related stimuli would serve adaptive purposes, such as curbing excessive exploratory behavior.

## Highlights

• Judgment bias induced in macaques by predator-related stimuli was examined
• Macaques evaluate ambiguous situations pessimistically after viewing snake pictures
• This judgment bias may help reduce risk-taking in potentially dangerous situations

## Graphical abstract

**Figure:**
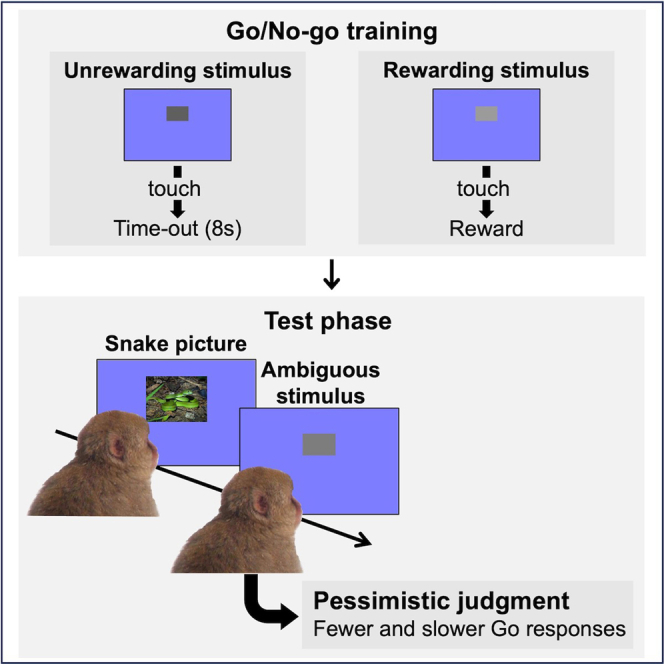

## Introduction

Affective states are inextricably tied to animals’ cognitive and behavioral propensities, serving as a vital adaptive response system to various challenges encountered in their natural habitats.^1^^,^^2^^,^^3^ The measurement and identification of the affective states that certain conditions elicit in animals will aid in clarifying the mechanisms and adaptive functions of affective response systems. The significance of accurately determining the affects experienced by animals in a given housing environment is increasingly recognized from an animal welfare perspective.^4^^,^^5^ However, discerning animals’ positive or negative affective valence based on their behavioral and physiological indicators is often challenging, as divergent affective states can give rise to equivalent behavioral or physiological states.^6^ Recently, researchers have utilized the judgment bias test to gauge animals’ affective valence based on the cognitive tendency that causes individuals with positive affects to expect favorable outcomes in ambiguous situations (i.e., optimistic bias) and those with negative affects to expect undesirable outcomes (i.e., pessimistic bias).^7^ This paradigm has been extensively employed in animal welfare and pharmacological research, where researchers estimate animals’ affective valence in response to enriched housing conditions or stressful interventions (for reviews, see^4^^,^^8^) as well as various pharmacological interventions (for a review, see^9^).

Predator-related stimuli represent crucial ecological information that directly impacts an animal’s survival. Such stimuli have been shown to elicit innate fear in prey animals and trigger specific cognitive and behavioral responses, such as rapid detection and avoidance.^10^^,^^11^^,^^12^ The cognitive and behavioral responses of primates, including humans, to snakes have been a topic of significant interest, considering the evolutionary pressure they face from such predators.^13^ Despite the abundance of research on this topic,^13^^,^^14^^,^^15^^,^^16^ the examination of biases in judgment potentially caused by predator-related stimuli has received limited attention (see^17^ for an exceptional study that evaluated the judgment bias induced in honeybees by a brief period of vibration, which the authors considered as a proxy for a predator attack). The lack of extensive examination of the judgment biases triggered by ecologically relevant stimuli, such as predator-related stimuli, hinders our understanding of these biases’ role in facilitating the adaptation of animals within their natural habitats (but see^18^ for a study that showed that species-specific behavior, i.e., tool use, in New Caledonian crows causes an optimistic judgment bias). Anticipating unfavorable outcomes in ambiguous situations after perceiving predator-related stimuli may be adaptive because it allows individuals to reduce risk-taking. Therefore, we hypothesized that Japanese macaques (*Macaca fuscata*) exhibit a pessimistic judgment bias after being exposed to a snake-related stimulus.

**Figure 1:**
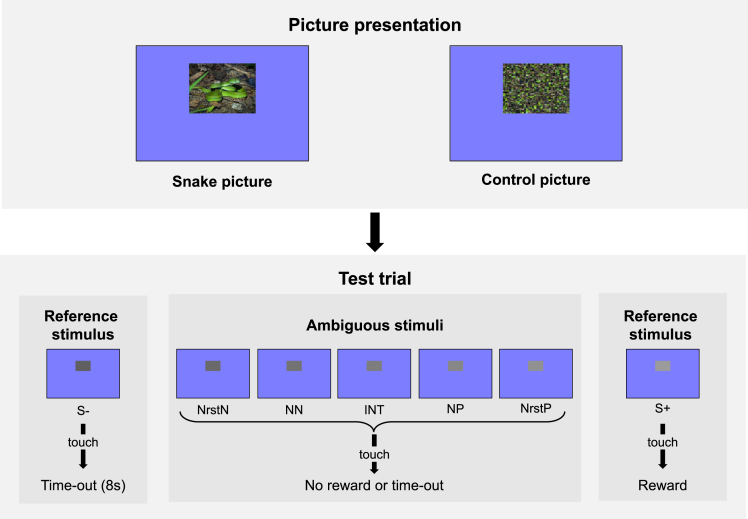
The experimental protocol Each session comprised 14 picture presentations and 14 test trials, with a picture presentation preceding each test trial. We adopted a block design, exclusively utilizing either snake or control pictures within a single session. Our stimulus set consisted of 28 snake pictures and 28 control pictures, which were generated by randomly scrambling the snake pictures. On each test trial, a single grayscale stimulus, selected from seven types of test stimuli (two trained reference stimuli [S+ and S−] and five ambiguous stimuli [NrstP, NP, INT, NN, and NrstN]), was presented. Each type of test stimulus was utilized twice in each session in a counterbalanced and pseudorandomized order. The allocation of dark and light tones to S+ and S− was counterbalanced across subjects.

Our study employed a within-subject design, where n = 8 captive Japanese macaques were subjected to a touchscreen-based Go/No-go judgment bias test. First, the subjects were trained to differentiate their responses depending on the luminance of square stimuli, either bright or dark. The stimulus was presented on the screen until the subjects touched it or 2000 ms passed. Correct Go responses to the square stimuli designated as positive stimuli (S+) were rewarded, while incorrect Go responses to negative stimuli (S−) were mildly punished (i.e., an 8000-ms time-out). Six of the eight subjects met the learning criteria (see STAR Methods) and were given 20 test sessions. In the test sessions, snake and control conditions were alternated every experimental day. Each session consisted of 282 trials, including 268 reference stimuli (S+ and S−) trials and 14 test trials, interspersed with either 14 snake picture presentations or 14 control picture presentations, depending on the condition (details provided in STAR Methods). The control pictures were generated by randomly scrambling a snake picture. The test stimuli comprised S+, S−, and the following five intermediate, ambiguous gray square stimuli: nearest positive (NrstP), near positive (NP), intermediate (INT), near negative (NN), and nearest negative (NrstN) (Figure 1). If individuals have a pessimistic judgment bias, they would hesitate to interpret an ambiguous gray stimulus as rewarding, resulting in fewer and slower Go responses. As an index of the judgment bias, we recorded the response time to the test stimulus. In order to integrate the subject’s no response into this index, we recorded the response time as 2000 ms (i.e., the maximum presentation duration) if the subject did not touch the test stimulus.^19^^,^^20^ We expected that subjects, after seeing pictures of snakes, would have a lowered expectation of favorable outcomes when faced with ambiguous situations. Hence, we predicted that the response time to the ambiguous stimuli would increase in the snake condition relative to the control condition.

## Results

**Figure 2:**
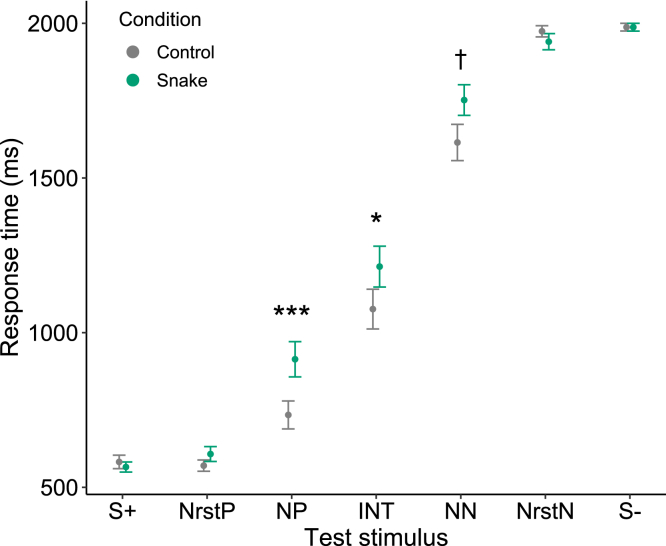
The mean response time according to the condition and the type of test stimulus Error bars represent standard errors. Sample size: n = 1680. Asterisks denote a significant interaction term between the condition and the type of test stimulus. ∗∗∗p < 0.001, ∗p < 0.05, †p < 0.1 (GLMM with a Gamma error structure).

**Figure 3:**
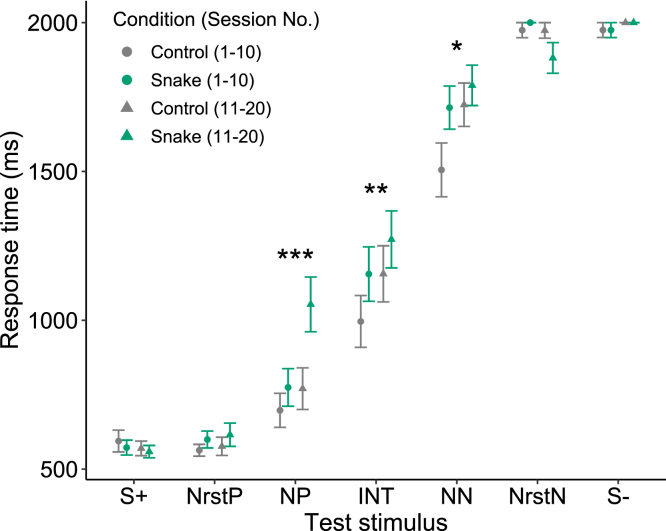
The mean response time according to the condition, session number, and type of test stimulus Error bars represent standard errors. For the purpose of illustration, we grouped the response times from the first ten sessions and the last ten sessions separately. Sample size: n = 1680. Asterisks denote a significant interaction term between the session number and the type of test stimulus. ∗∗∗p < 0.001, ∗∗p < 0.01, ∗p < 0.05 (GLMM with a Gamma error structure).

**Figure 4:**
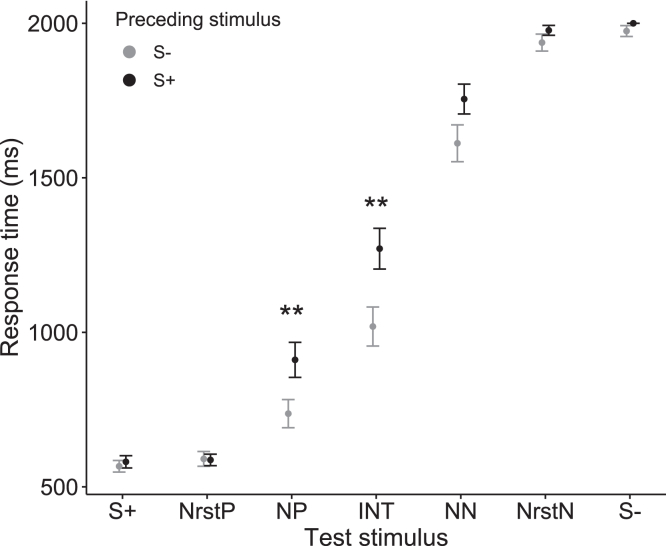
The mean response time according to the type of test stimulus and type of preceding stimulus Error bars represent standard errors. Sample size: n = 1680. Asterisks denote a significant interaction term between the type of preceding stimulus and the type of test stimulus. ∗∗p < 0.01 (GLMM with a Gamma error structure).

Our results demonstrated that Japanese macaques exhibited a pessimistic evaluation of ambiguous stimuli when exposed to pictures of snakes. Our statistical model (see STAR Methods) revealed that response times to NP and INT were significantly prolonged after viewing the snake pictures compared to the control pictures (GLMM: Condition × test stimulus [NP], β = 0.241 ± 0.07, p = 0.0006; Condition × test stimulus [INT], β = 0.163 ± 0.071, p = 0.0208; Figure 2; Table S1). Additionally, the results suggest that the response time to NN tended to be longer after viewing the snake pictures, although this did not reach statistical significance (GLMM: Condition × test stimulus [NN], β = 0.118 ± 0.071, p = 0.0948; Figure 2; Table S1). The statistical model also revealed that the response times to NP, INT, and NN increased with session number (GLMM: session number × test stimulus [NP], β = 0.022 ± 0.006, p = 0.0004; session number × test stimulus [INT], β = 0.017 ± 0.006, p = 0.0082; session number × test stimulus [NN], β = 0.013 ± 0.006, p = 0.0287; Figure 3; Table S1). Furthermore, our results indicate that the response times to NP and INT were slower when the preceding stimulus was S+ compared to when it was S−, suggesting that the contrast between the immediately preceding unambiguous reference stimulus and the subsequent ambiguous stimulus has an impact on the response time (GLMM: preceding stimulus [S+] × test stimulus [NP], β = 0.184 ± 0.071, p = 0.0096; preceding stimulus [S+] × test stimulus [INT], β = 0.191 ± 0.071, p = 0.0073; Figure 4; Table S1).

## Discussion

The increased response time to ambiguous stimuli in the snake condition should not be interpreted as due to prolonged immobility or overall reaction speed decline triggered by the snake pictures. This is because the response time to trained unambiguous stimuli (S+ and S−) did not vary between conditions. Similarly, the response times to stimuli nearest S+ and S− (NrstP and NrstN, respectively) remained the same between conditions, likely due to their similarity in luminance to the trained reference stimuli. These results indicate that the exposure to the snake pictures did not distort the subjects’ overall color perception but instead amplified their anticipation of an unfavorable outcome in ambiguous situations and/or reduced their expectation of a positive outcome. Furthermore, our results that subjects’ reaction speed decreased exclusively to ambiguous stimuli rule out the possibility that an attentional shift away from threat stimuli in animals experiencing negative affect, as some studies have shown,^21^ caused an overall delay in approaching the test stimuli on the screen. Previous studies have shown that humans in a depressed mood^22^ and rats in a depression-like state caused by chronic stress^23^ tend to exhibit negative expectations concerning future events. Our within-subject comparisons demonstrate that even brief exposure to predator-related stimuli can induce a similar pessimistic bias. Unlike prior research that has shown that anxious chicks avoided ambiguous shapes resembling predators^24^ and that Japanese tits that heard snake-specific alarm calls inspected sticks moving like snakes,^25^ our study demonstrates that exposure to predator-related stimuli can engender pessimistic judgments not only in predator-related circumstances but also in ambiguous situations in general.

Importantly, the judgment bias induced by the snake pictures was not eliminated by the confounding learning effect through repeated testing. Our findings revealed that the subjects’ response times to ambiguous stimuli slowed down over the course of successive sessions. This result suggests that the subjects may have acquired (although not entirely) the understanding that ambiguous stimuli are not predictive of rewards. This outcome is consistent with several studies that have indicated that the impact of learning on animals’ responses to ambiguous stimuli can represent a significant confounding factor that affects the interpretation of results from judgment bias tests.^19^^,^^26^^,^^27^ It is worth mentioning that we detected a learning effect despite having reduced the ratio of ambiguous stimulus trials relative to reference stimulus trials in each session (274:10) and having implemented a variable reinforcement schedule (80%) following prior research.^19^^,^^20^^,^^28^ Nevertheless, despite the learning effect, the tendency for response times to be prolonged in the snake condition relative to the control condition was persistent even in later trials, as depicted in Figure 3. Additionally, the absence of a learning effect for NrstP and NrstN, which were closest to the reference stimuli, further supports our interpretation that no judgment bias was detected for these stimuli as they were perceived as virtually indistinguishable from the reference stimuli.

In conclusion, the present study has demonstrated that Japanese macaques exposed to pictures of snakes exhibited a pessimistic judgment bias. By demonstrating that animals hesitate to approach ambiguous stimuli after encountering predator-related information, our finding raises the possibility that this judgment bias may have evolved as an adaptive response to deal with potential threats in natural habitats. In environments characterized by high levels of threat^29^^,^^30^ or, more broadly, where the adage of “misfortunes never come singly” holds true, behavioral strategies that mitigate risk-taking would be evolutionarily advantageous. Hence, an affective response system that lowers expectations of favorable outcomes when faced with ambiguous situations after encountering threat-related stimuli would serve an adaptive purpose, such as curbing excessive exploratory behavior.

### Limitations of the study

Future research should address several limitations and lingering questions present in this study. Firstly, exploring the judgment bias engendered by stimuli related to predators other than snakes would also be a pertinent area of inquiry. For example, raptors, carnivores, and spiders could also pose a threat to primates.^31^^,^^32^ It would be interesting to examine whether stimuli related to other types of predators evoke a pessimistic bias and determine its relative magnitude. Secondly, future research should examine the impact of the subjects’ prior experiences with predators on the magnitude of the bias. The subjects in this study were either born in indoor cages or group-housed outdoor enclosures. The latter individuals may have encountered snakes while residing in the outdoor enclosures, thereby affecting their response to the snake pictures. Nevertheless, even a subject born and raised in indoor cages, who should not have had any experience with snakes, showed a comparable pessimistic bias in response to snake pictures, implying that this response may be innate. This is consistent with previous research suggesting that snakes are innately fear-provoking stimuli for primates.^16^ A further investigation into the influence of individuals’ prior experiences with snakes on their decision-making is imperative for a better understanding of the mechanisms and adaptive functions of animals’ affective response systems. Additionally, a deeper understanding of how predator-related stimuli shape the behavior of animals in their natural habitats is essential to unraveling the ecological significance of judgment biases. Moreover, the present study’s finding that presenting predator-related stimuli as an acute stressor lowers expectations of favorable outcomes is in contrast to a study that showed chronic stress lowers animals’ vigilance, resulting in a faster approach to food rewards.^21^ Investigating whether ecologically relevant negative stimuli, such as predator-related acute stressors, and long-term poor enrichment conditions, such as individual housing and rest deprivation,^21^ have differential impacts on expectations of future outcomes and attention biases would be an interesting area for future research. Furthermore, elucidating judgment biases induced by other types of ecologically relevant stimuli (e.g., social stimuli;^33^ species-specific behaviors^18^) is important for a comprehensive understanding of the adaptive function of an affective response system.

## STAR★Methods

### Key resources table

**Table:** 

REAGENT or RESOURCE	SOURCE	IDENTIFIER
**Deposited data**		

Data	This paper	Mendeley Data: https://doi.org/10.17632/ddntr48m83.2

**Experimental models: Organisms/strains**		

Japanese macaques (*Macaca fuscata*)	Center for the Evolutionary Origins of Human Behavior, Japan	N/A

**Software and algorithms**		

R	R Foundation	https://www.r-project.org/

### Resource availability

#### Lead contact

Further information and requests should be directed to and will be fulfilled by the lead contact, Sakumi Iki (sakumi.iki@gmail.com).

#### Materials availability

This study did not generate new unique reagents.

### Experimental model and study participant details

Eight adult Japanese macaques, consisting of three males and five females aged 7 to 18 years (13.25 ± SD 3.42 years old), were tested at the Center for the Evolutionary Origins of Human Behavior (EHUB), Kyoto University. The macaques were housed either singly or in pairs, with partitioning implemented in the latter case at least 30 minutes before the experiment to separate them. In order to control motivation for reward, the subjects were not fed before the trial. Upon completion of the experiment, the subjects were given daily rations of 450 grams of monkey chow (AS, Oriental Yeast Co., Ltd.) and a thrice-weekly ration of radish and carrot. Access to water was made available *ad libitum*. This study was approved by the Animal Welfare and Care Committee of the EHUB, Kyoto University (#2022–103).

### Method details

#### Apparatus

The picture stimuli (560 × 420 pixels) and the reference and ambiguous stimuli (300 × 200 pixels) were displayed via a touchscreen (Microsoft Surface Pro 8 EIV-00010). The touchscreen was positioned in front of each monkey’s cage, granting them an unobstructed view of the screen. A food dispenser (Med Associates, Fairfax, Vermont, USA) automatically dispensed a banana-flavored pellet (Bio-Serv DPP 190 mg) from the tray beneath the screen upon a correct response, accompanied by a one-second beep. A buzzing signal accompanied an incorrect response. The experimental apparatus was controlled using Visual Basic 2010 (Microsoft Corporation, Redmond, Washington, USA). The control pictures were generated using a Python script, which divided a picture of a snake into 32 × 32 rectangles and scrambled them randomly, resulting in 28 control pictures from 28 snake pictures. The control pictures, therefore, possessed the same low-level image characteristics, such as luminosity, color, and size, as their snake counterparts. The same methodology was employed to create randomly scrambled neutral artificial object pictures used in the training phase.

#### Training phase

Subjects underwent training in a Go/No-go visual discrimination task from June to December 2022, with subsequent testing carried out from December 2022 to January 2023. As reference stimuli, three dark gray (RGB 75/75/75, 85/85/85, and 95/95/95) and three light gray stimuli (RGB 155/155/155, 165/165/165, and 175/175/175) were employed, serving as predictors of reward (S+) and predictors of mild, non-invasive punishment (S-). To prevent subjects from developing a habit of approaching only one point along the grayscale, three different gray tones of slightly varying darkness were assigned to S+ and S-, respectively. The allocation of dark and light tones to S+ and S- was counterbalanced between subjects. S+ and S- stimuli were displayed in the upper center of the screen for 2 seconds after the subjects touched the self-start key located in the lower center of the screen. The order of S+ and S- stimuli was pseudorandomized, with all six reference stimuli appearing every six trials. At the start of each training session, S+ was always presented. When a subject touched S+, the stimulus disappeared, and a reward was delivered. An 80 % variable reinforcement ratio was implemented to mitigate the learning effects of unrewarding ambiguous stimuli.^19^^,^^20^ The inter-trial interval (ITI) was 2000 ms. In response to a touch on S-, the stimulus disappeared, and the subject was required to wait an 8000 ms time-out in addition to the ITI before proceeding to the next trial. Training sessions were conducted every weekday.

The training protocol consisted of two distinct stages. In the first stage, subjects participated in as many S+ and S- discrimination trials as possible within one hour. Following each day’s training, we computed the proportions of correct Go and No-go responses. Subjects who completed at least 100 training trials per day and whose proportions of correct Go and No-go responses were 0.9 or greater for at least three consecutive days were eligible to proceed to the subsequent training stage.

The second training stage was designed to acclimate subjects to the presentation of pictures, as mere exposure to pictures, even in the absence of harmful content, may elicit adverse affective responses from subjects who have not previously encountered picture stimuli on a touchscreen. This stage of training was executed as follows:

1. 40 trials of the discrimination task were conducted, with S+ and S- presented an equal number of times.
2. 10 trials of the discrimination task were conducted, with S+ and S- presented an equal number of times, followed by the presentation of either a neutral picture (i.e., a picture of an artificial object) or a randomly scrambled picture of an artificial object in the upper center of the screen for 1 second.
3. The procedure described in step (2) was repeated 18 times, alternating between pictures of neutral objects and scrambled pictures. The picture was pseudorandomly selected from a dataset of 20 pictures (ten pictures of neutral artificial objects and ten scrambled pictures).
4. 10 trials of the discrimination task were conducted, with S+ and S- presented an equal number of times.

Subjects who completed all steps (1) through (4) and whose proportions of correct Go and No-go responses were 0.9 or greater for at least three consecutive days proceeded to the testing phase. Two of the eight subjects failed to meet the learning criteria within the designated training period and were thus unable to move on to the testing phase.

#### Testing phase

Two dark gray (RGB 85/85/85 and 95/95/95) and two light gray stimuli (RGB 155/155/155 and 165/165/165) were employed as S+ and S-. To increase the difficulty in distinguishing between the ambiguous and reference stimuli, reference stimuli furthest from the ambiguous stimuli (RGB 75/75/75 and 175/175/175) were omitted from the testing phase. Ambiguous stimuli were comprised of five intermediate gray stimuli (RGB 105/105/105, 115/115/115, 125/125/125, 135/135/135, and 145/145/145), which were labeled as Nearest Positive (NrstP), Near Positive (NP), Intermediate (INT), Near Negative (NN), and Nearest Negative (NrstN) based on their proximity to the S+ and S- stimuli. Testing was conducted on weekdays, employing a block design experiment alternating between snake and control conditions every day, with ten sessions in each condition. The order of the snake and control conditions was counterbalanced across subjects. The 28 snake pictures were divided into two sets of 14 pictures each, and likewise, the 28 control pictures were divided into two sets of 14 corresponding pictures each. Each test session consisted of 14 times of picture presentation and utilized one of the picture sets. The first set of 14 snake pictures and its corresponding set of 14 control pictures were used alternately in two consecutive test sessions, and then the other sets of snake pictures and control pictures were used alternately in the next two test sessions. We repeated this procedure five times since our experiment comprised ten sessions per condition. The set of pictures utilized in the first test session was selected pseudorandomly. The testing phase was conducted as follows:

1. 40 filler trials of the discrimination task were conducted, with S+ and S- presented an equal number of times. After completing the 40 trials, the proportions of correct Go and No-Go responses were calculated. If a subject’s proportions of correct responses were 0.9 or greater, they proceeded to the next step. If the proportions were less than 0.9, an additional 110 trials of the discrimination task were performed, and the subject did not advance to the subsequent steps for that day.
2. 20 filler trials of the discrimination task were conducted, interspersed with two presentations of neutral pictures (one picture of an artificial object and one randomly-scrambled picture). The presentation of pictures occurred after every ten trials. The pictures were selected in a counterbalanced order from the same stimulus set used in the second training stage. This step was introduced to prepare the subjects for the picture presentation, as they might be startled by the sudden presentation itself, regardless of the content of the picture.
3. 10 filler trials of the discrimination task were conducted, where the reference stimuli with RGB values of 85/85/85 and 165/165/165 were shown three times each, and 95/95/95 and 155/155/155 were shown twice each, presented in a pseudorandom order.
4. Either a snake or a control picture was presented, dependent on the condition of the day.
5. Two filler trials of the discrimination task were conducted, with one trial each of S+ and S- (RGB 95/95/95 and 155/155/155) presented in an order counterbalanced within and across subjects. We introduced these two filler trials to allow the subjects to confirm that touching the self-start key would lead to the presentation of the gray stimuli, not to the presentation of snake/control pictures.
6. One test trial was conducted, presenting one of the following seven test stimuli for 2000 ms: S+, S- (RGB 95/95/95 and 155/155/155), or one of the five types of ambiguous stimuli. If subjects touched the ambiguous stimulus, it disappeared; the ambiguous stimulus did not predict either a reward or mild punishment. The response time to the test stimulus was recorded.^20^^,^^21^
7. Steps from (3) to (6) were repeated 14 times. Since we adopted a block design experiment, we exclusively presented either 14 snake pictures or 14 control pictures within a single session. Each of the seven test stimuli was presented twice in each session. All seven test stimuli were used in the first half of these 14 repetitions. The seven test stimuli were presented in the second half of the repetition in the same order as the first half. The order of the test stimuli was counterbalanced within and across subjects. The presentation of test stimuli and their combination with the preceding snake or control picture was also counterbalanced within and across subjects.
8. 40 filler trials of the discrimination task were conducted, with S+ and S- presented an equal number of times.

An 80% variable reinforcement ratio was implemented in steps (1), (2), (3), and (8). Over the course of 20 sessions, each of the seven test stimuli was presented to each subject 20 times in both the control and snake conditions, yielding a total of 1680 (= 7 test stimuli [S+, S-, five ambiguous stimuli] × 2 presentations per session × 10 sessions per condition × 2 conditions [snake, control] × 6 subjects) recorded response times to the test stimuli.

### Quantification and statistical analysis

We analyzed the data utilizing Generalized Linear Mixed Models (GLMMs) with the glmer function in the lme4 package in R version 4.1.2.^34^ We set our alpha level to 0.05.

A GLMM with a Gamma error structure and a log link function was employed to analyze the response time to the test stimuli. We included the condition (categorical: snake, control), the type of test stimulus (categorical: S+, S-, NrstP, NP, INT, NN, NrstN), and a two-way interaction between the condition and the type of test stimulus as key predictors. To control for potential confounding effects, the following factors were included as control variables: session number (continuous: Day 1-20), the type of learned stimulus preceding the test stimulus (categorical: S+, S-), a two-way interaction between the type of test stimulus and session number, and a two-way interaction between the type of test stimulus and the type of preceding stimulus. Subject’s sex (categorical) and birthplace (categorical: indoor, outdoor) were also included as control variables. We dealt with pseudoreplication by including subject ID as a random intercept and a random slope for the type of test stimuli. The significance of the model was confirmed through comparison with a reduced model omitting the condition (snake/control) but including other variables, a random intercept, and a random slope, and a null model with only a random intercept, utilizing a likelihood ratio test with the anova function (Table S2). The proportion of total variance explained by the model was determined by calculating the marginal R^2^, which accounts for variance explained by fixed factors, and the conditional R^2^, which accounts for variance explained by both fixed and random factors, using the r.squaredGLMM function in the MuMIn package^35^ (Table S2).

## Data Availability

• All data and code have been deposited at Mendeley Data: https://doi.org/10.17632/ddntr48m83.2. • This paper does not report original code. • Any additional information required to reanalyze the data reported in this paper is available from the lead contact upon request.
