# Supplementary material for: Fearful snake pictures make monkeys pessimistic
Source: iScience. 2023 Aug 12;26(9):107622. doi: 10.1016/j.isci.2023.107622 (PMC10474457; doi:10.1016/j.isci.2023.107622)
Supplement: Document S1. Tables S1 and S2 [file mmc1.pdf]

iScience, Volume *26*

Supplemental information

Fearful snake pictures make monkeys pessimistic

Sakumi Iki and Ikuma Adachi

Table S1. Results of the GLMM analysis, related to Figures 2, 3, and 4.

	β	SE	t value	p value
Intercept	6.2933	0.1036	60.7570	<0.0001***
Condition (snake)	-0.0257	0.0498	-0.5159	0.6059
Test stimulus (NrstP)	-0.0419	0.0878	-0.4777	0.6329
Test stimulus (NP)	-0.1027	0.1072	-0.9581	0.3380
Test stimulus (INT)	0.3189	0.1205	2.6462	0.0081*
Test stimulus (NN)	0.8322	0.1166	7.1365	<0.0001***
Test stimulus (NrstN)	1.2231	0.1083	11.2947	<0.0001***
Test stimulus (S-)	1.1933	0.1104	10.8089	<0.0001***
Session number	-0.0040	0.0043	-0.9469	0.3437
Preceding stimulus (S+)	0.0379	0.0501	0.7567	0.4492
Sex (male)	-0.0596	0.0379	-1.5712	0.1161
Birthplace (outdoor)	0.1254	0.0820	1.5302	0.1260
Condition (snake) × Test stimulus (NrstP)	0.0857	0.0704	1.2164	0.2238
Condition (snake) × Test stimulus (NP)	0.2414	0.0705	3.4228	0.0006***
Condition (snake) × Test stimulus (INT)	0.1631	0.0706	2.3118	0.0208*
Condition (snake) × Test stimulus (NN)	0.1177	0.0705	1.6708	0.0948†
Condition (snake) × Test stimulus (NrstN)	0.0078	0.0704	0.1114	0.9113
Condition (snake) × Test stimulus (S-)	0.0257	0.0704	0.3650	0.7151
Session number × Test stimulus (NrstP)	0.0043	0.0061	0.7141	0.4752
Session number × Test stimulus (NP)	0.0218	0.0062	3.5420	0.0004***
Session number × Test stimulus (INT)	0.0165	0.0063	2.6430	0.0082**
Session number × Test stimulus (NN)	0.0133	0.0061	2.1871	0.0287*
Session number × Test stimulus (NrstN)	0.0019	0.0061	0.3094	0.7570
Session number × Test stimulus (S-)	0.0057	0.0061	0.9392	0.3476
Preceding stimulus (S+) × Test stimulus (NrstP)	-0.0398	0.0706	-0.5642	0.5726
Preceding stimulus (S+) × Test stimulus (NP)	0.1837	0.0709	2.5910	0.0096**
Preceding stimulus (S+) × Test stimulus (INT)	0.1906	0.0710	2.6846	0.0073**
Preceding stimulus (S+) × Test stimulus (NN)	0.0810	0.0710	1.1405	0.2541
Preceding stimulus (S+) × Test stimulus (NrstN)	-0.0173	0.0706	-0.2456	0.8060
Preceding stimulus (S+) × Test stimulus (S-)	-0.0248	0.0706	-0.3518	0.7250

Sample size: n = 1680 trials. Subject ID was included as a random intercept and a random slope for the type of test stimuli.

Table S2. Details of the model comparison, related to STAR Methods.

	Model	AIC	logLik	Chisq	Df	Pr(>Chisq)
Null	RT~ 1 + (1|ID)	26672	-13333			
Model w/o condition	RT~ SN*TS + PS*TS + S + BP+ (1+TS|ID)	25076	-12486	1694.83	49	<0.0001
Full	RT ~ C*TS + SN*TS + PS*TS + S + BP + (1+TS|ID)	25058	-12470	31.22	7	<0.0001

ID: subject ID; RT: response time; SN: session number; TS: type of test stimulus; C: condition S: sex; BP: birthplace. The full model accounted for significantly more variance than the model without the condition and the null model (Likelihood ratio test: $p < 0.0001$). The fixed factors of the full model explained 61.2% of the variance (i.e., Marginal $R^2 = 0.612$), and the fixed and random factors of the full model explained 62.4% of the variance (i.e., Conditional $R^2 = 0.624$).
